# Lessons learned from unsuccessful use of personal carbon monoxide monitors to remotely assess abstinence in a pragmatic trial of a smartphone stop smoking app – A secondary analysis^☆^

**DOI:** 10.1016/j.abrep.2018.07.003

**Published:** 2018-07-23

**Authors:** Aleksandra Herbec, Jamie Brown, Lion Shahab, Robert West

**Affiliations:** a Department of Behavioural Science and Health, University College London, London, UK; b UCL Tobacco and Alcohol Research Group (UTARG), UK

**Keywords:** Smoking cessation, Intervention, Smartphone, App, Pilot, Carbon monoxide, Biochemical verification

## Abstract

**Introduction:**

Verifying abstinence remotely in trials of digital cessation interventions remains a major challenge. This study reports on using personal carbon monoxide (CO) monitors to assess abstinence in a pragmatic trial of a standalone cessation app involving automated recruitment with no researcher contact.

**Methods:**

The study involved secondary data analysis of remote CO testing in a randomized trial (ISRCTN10548241) comparing two versions of a cessation app (BupaQuit). Trial participants were adult UK-based smokers interested in quitting, who were recruited online (02/2015–03/2016). Participants were followed-up through the app, email or phone at 4 weeks. Fifty-nine participants reporting not smoking were posted a personal CO monitor with instructions, and emailed two reminders. The monitors required installing software on a Windows PC. Participants were not reimbursed but retained the device. We recorded the proportion of CO tests returned, test results, self-reported ease of use, correct use, acceptability, and reasons for missing results.

**Results:**

Fifteen (25.4%) CO results were returned, of which 86.6% were <10 ppm and 53.3% were <5 ppm, indicating abstinence (corresponding to 20.9% and 12.9% of all trial participants self-reporting abstinence, respectively). These 15 participants found the test easy, acceptable and believed they conducted it correctly. Eight (18.2%) of the missing results were accounted for, including no access to a Windows PC, barriers to receiving packages, and unwillingness to share results.

**Conclusion:**

Remote validation using personal CO monitors may not yet be feasible in pragmatic studies of cessation apps in which participants are recruited with no reimbursement or direct contact with researchers.

## Highlights

• Conducting remote verification of smoking abstinence remains a challenge in trials.
• Personal carbon monoxide (CO) monitors could be posted to trial participants.
• Only 25% of participants reporting abstinence returned their CO test results.
• Remote testing with personal PC-connected CO monitors emerged as infeasible.
• Lack of reimbursement and contact with researchers could contribute to the findings.

## 1 Introduction

Verification of self-reported abstinence is important in evaluating cessation interventions (Benowitz et al., 2002; West et al., 2005). However, it is especially challenging in studies of digital interventions that rely on remote recruitment and follow-up, with participants often spread across vast geographical locations. In result, many of such studies rely on self-reports (Taylor et al., 2017; Ubhi et al., 2015; Whittaker et al., 2016), which may over-estimate the actual quit rates (West et al., 2007), although the bias may be lower in low-intensity interventions, and should not differ across study arms (Glasgow et al., 1993; Patrick et al., 1994). In this study we report findings from using one type of a personal carbon monoxide (CO) monitor, which connected to Windows computers, to remotely validate self-reported abstinence in a trial of a smartphone stop smoking app. The trial involved no contact with the researchers during enrolment, and only minimal contact at follow-up.

Several methods for remote verification of abstinence are available. One approach involves analysing samples of saliva for nicotine metabolites (cotinine or anabasine). These can be collected through post, and sent by participants to the researchers or directly to biochemical labs (Brown et al., 2014). Previous studies offered participants reimbursement for providing saliva samples (e.g. reimbursement of value of £20/$28) (Brown et al., 2014). The costs of conducting lab-based tests of saliva samples can be at least £25–£40/$34–$55 per participant, excluding reimbursement (in 2015, the costs of cotinine tests started at £20–£35 per sample, depending on the level of processing required, over £30 for anabasine tests, and around £5 for first class postage, envelops and salivettes; the prices could change depending on lab and the number of tests, and certain items may need to be purchased in bulk, e.g. salivettes). However, saliva testing is not suitable when researchers have no access to a suitable laboratory for testing. New saliva testing kits might enable remote home-based testing, with participants sending the results as photos or live video (Marrone et al., 2010). Remote assessment of heart rate variability through a smartphone camera has not been validated yet, but it could offer another, potential cost-effective and convenient method (Harte & Meston, 2014; Heathers, 2013; Peng et al., 2015).

Assessment of carbon monoxide (CO) in the exhaled breath has been among the most commonly used methods in many cessation programmes (Goldstein et al., 2018; West et al., 2010), with readings lower than 10 particles per million (ppm) commonly accepted as confirmation of abstinence (Brose et al., 2013; West et al., 2005), but with lower cut-off levels of 5 ppm suggested more recently (Perkins, Karelitz, & Jao, 2013). CO testing has important advantages over the other methods as it is non-invasive, and insensitive to concurrent use of nicotine products or e-cigarettes, although its temporal applicability is limited due to rapid elimination of CO from the body (Benowitz et al., 2002; Goldstein et al., 2018). Measuring CO levels may be especially difficult if participants cannot travel for in-person testing. Some studies have accomplished remote CO testing by having research staff travel to participants' homes or organizing verification at local clinics (Kim et al., 2005), or by providing traditional CO monitors for home-based testing and requiring participants to share videos of the procedure (Dallery & Glenn, 2005; Hertzberg et al., 2013; Karelitz et al., 2017). However, such a method may not be feasible or affordable in many contexts.

A new generation of personal CO monitors (e.g. devices manufactured by Bedfont® Scientific Ltd., https://www.bedfont.com/shop/smokerlyzer) are smaller, lighter and more affordable (under £50/$69 in the UK) than traditional CO monitors (cost starting at around £170/$230 in the UK), which means they could be purchased and posted to smokers for home-based testing at a price similar to the saliva testing that includes reimbursement.

Remote CO testing using these new devices offers several advantages. CO testing is not easily accessible, but has been shown to be valued by smokers (Beard & West, 2012; Goldstein et al., 2018; Shahab, West, & McNeill, 2011), and acceptable for regular remote assessment of smoking status (McClure et al., 2015), and thus could be attractive to study participants, enabling them to assess their quitting progress. Retaining such a device for future use could be a form of compensation for participants' time and inconvenience, especially in the absence of other reimbursement. Importantly, a single CO device could be used for multiple tests and follow-up waves. Moreover, CO testing in itself may provide an incentive for smokers to remain abstinent (Beard & West, 2012; Shahab et al., 2011). Finally, using the new generation of personal CO monitors could be cost-effective, and appropriate when saliva testing or use of traditional CO monitors is not possible. However, feasibility of using this method to assess self-reported abstinence during follow-up in trials of digital interventions is yet to be ascertained.

In this study we assessed feasibility of remote verification of abstinence using personal CO monitors that connect to Windows PCs, and which were posted to participants who were self-reporting not smoking in a pragmatic trial of a stop smoking smartphone app (BupaQuit). One of the key underlying aims for the BupaQuit trial was to evaluate the app in a more ecological setting than previous studies had done (Bricker et al., 2014; Bricker et al., 2017; Buller et al., 2014), namely one with limited contact with the researcher throughout the trial. The procedures of CO testing were aligned with those used in an earlier trial of a web-based stop smoking intervention conducted by some of the authors, and which involved saliva sampling (Brown et al., 2014). These included automatic posting of the testing kit to the postal address provided by the participants during enrolment into the trial, using first class mail, and offering guidance to participants in the form of written instructions. The main difference in the procedures was the lack of incentives in the current trial beyond the possibility to keep the CO device for private use. Adopting similar procedures for CO testing was judged to be important - if CO testing was to be a feasible alternative to saliva testing, it should be used it in a context with similar funding level and research staff time.

## 2 Methods

### 2.1 Design

The study involved secondary analysis of data related to remote CO testing from a pragmatic, randomized controlled trial comparing effectiveness of a cessation app for iOS and Android called BupaQuit, to a version of the app providing minimum support. The trial was approved by UCL Research Ethics Committee (6212/001), and was prospectively registered with the ISRCTN Register (ISRCTN10548241). The trial outcomes are reported elsewhere (submitted for review). Trial protocol and additional information are available on Open Science Framework (https://osf.io/ge6vh/). The study was conducted in collaboration with Bupa (www.bupa.com) who developed the app and provided support for data collection.

### 2.2 BupaQuit trial recruitment and participants

Between February 2015 and March 2016, we recruited into BupaQuit trial a total of 425 adult, UK-based daily smokers interested to quit, who downloaded the app, and completed registration (including provision of informed consent and full contact details) via the app. Participants were recruited online and with no researcher contact, primarily through paid advertisement on social media, as well as through UK iTunes and Google Play app stores. Interested participants were directed to a project website with study information that outlined the follow-up procedures (including the follow-up procedures at 2 and 7 months), and asked to confirm reading it before registering via the app.

### 2.3 Study sample

Among BupaQuit trial sample, 62 (14.6%) participants self-reported not smoking in the past 14 days at 4-week follow-up (primary trial outcome). The current study concerned a sample of 59 trial participants who (i) self-reported not smoking, and (ii) were posted the CO monitor (three participants were not posted a monitor due to one participant declining to receive one, and the other two due to administrative reasons).

### 2.4 Procedure

Trial participants were contacted at 4-week follow-up since their quit date, first via the app, and then email and phone to assess the primary outcome. Participants self-reporting abstinence were posted a 1st Class small parcel (normally delivered on the next business day within the UK; CO box size 25 × 15 × 5 cm plus padded envelop; stamp cost starting at £3.30/$4.50) with the COmpactUSB™ Smokerlyzer® developed by Bedfont Scientific Ltd. (the only such device available for purchase at the time). The package included additional mouthpieces (enabling hygienic sharing of the device), and instructions and information about CO testing (see Appendix A1). Participants were instructed to provide a single CO reading upon receiving the CO device, and were also informed that they may be asked to use the device again at the next follow-up at 6 months. However, due to the low return rate of CO readings during the short-term follow-up the biochemical verification at 6 months was suspended.

Only those participants, who were reporting not smoking over the phone (66.1% of the current study sample) could be opportunistically asked to update the postal address. Most of the participants contacted over the phone seemed positive about the CO test, but a longer discussion about the procedures was not possible, and the participants often insisted to end the call due to being busy (internal communication within the research team). Participants who reported not smoking over the app or email were not engaged in additional communication to confirm the postal details. Our reasons were: (i) the re-contact rates via app and email to collect other outcome data were low, and it was judged unlikely that using these channels could enable efficient collection of additional information on postal address; (ii) such additional communication could over-burden participants, and negatively affect their responses to the 6 months follow-up; (iii) the original protocol approved by the ethics committee did not include additional communication for such purposes and would have required an amendment; (iv) the study aimed to re-create conditions reported in other trials attempting biochemical verification, e.g. through collecting saliva sample, that had not routinely confirmed contact details but relied on the information provided at baseline (Brown et al., 2014); attempting to confirm such details (v) would require additional resources and staff time, and (vi) would most likely delay posting the device, or even prevent it.

Using COmpactUSB™ Smokerlyzer® required participants to download a dedicated software for Windows PCs, also created and adapted for the trial by Bedfont® Scientific Ltd. (Fig. A1). This was a potential barrier to use, but it was expected that many participants would have access to a Windows PC at home or work (NetMarketShare, n.d.). Upon completing the CO test, participants could email the result from within the software directly to the researchers. Participants could take multiple tests and decide which of the results to email. Although this allowed participants to use the device without the study team's knowledge, it guarded against situations when multiple CO results would be available for the same participant (e.g. due to practicing, or device sharing), and to enable participants to use the device in private. Participants were sent one information email (with summary of instructions and a link to software download), and a reminder within a week. Participants were not reimbursed, but retained the device for personal use. Packages with CO devices were posted through Bupa internal postal services and were not tracked due to (i) high cost, and (ii) practical difficulties setting up tracking within the Bupa service.

### 2.5 Measures

Anonymised data were recorded and managed in Excel spreadsheets by AH and a team of trained research assistants, and used only for this study. The device manufacture had no access to the data.

#### 2.5.1 Baseline and process measures

We recorded participants' age, gender, occupation, education, study arm, follow-up channels through which participants were contacted (app, email, phone), as well as phone operating system (iOS, Android, or *Unknown* – due to BupaQuit database architecture, some participants had missing data on app usage and the operating system, thus were classified to Unknown. This data missingness could have resulted from genuine disengagement from the app, or failure of data synchronisation; these participants were included in the trial and in the present study).

#### 2.5.2 Outcome measures

We assessed: (a) proportion of CO tests returned, (b) time to receiving results (difference between dates of CO posting and test return), (c) proportion of tests confirming abstinence (<10 ppm; only the first result sent was considered), (d) test acceptability: *Did you find the CO monitor an acceptable way to assess your abstinence status?*, (e) ease of use: *Did you find the CO monitor easy to use?*, (f) correct use: *Do you think you managed to use the CO monitor properly?*. Data on items (c)–(f) were collected through the software. Additionally, reasons for missing CO results were opportunistically collected during future communication with trial participants, where possible. Table A1 in the Appendix lists other outcomes from BupaQuit trial for the current sub-sample, which are auxiliary to the present study.

### 2.6 Data analysis

Participants with and without the CO results returned were compared on baseline and process variables using chi-square and *t*-test for categorical and continuous data, respectively. We applied Sidak correction to account for multiple comparisons, and the *p*-value cut-off was set to 0.007. Data were analysed in SPSS (22.0).

## 3 Results

**Fig. 1:**
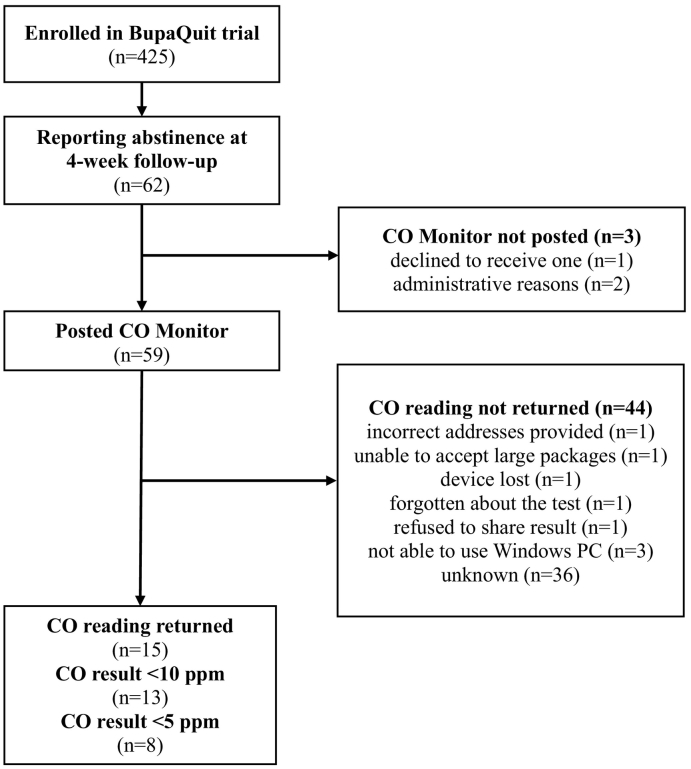
Participant flow through BupaQuit trial and CO testing procedure.

Fig. 1 presents the flow of participants. Fifteen out of 59 (25.4%) participants returned their CO readings (one participants sent two readings, and only the first reading was included in the analysis), of which five did so within two days, six within a week, and three after 9, 22 and 47 days since the device was posted (mean = 8.4 days, median = 5 days; for one participant the date was not available). Thirteen (86.6%) of the returned readings were below 10 ppm (meeting the Russel Standard criteria for abstinence (West et al., 2005)), and eight (53.3%) were below 5 ppm (a more conservative threshold suggested more recently (Perkins et al., 2013)). This corresponded to 20.9% and 12.9% of all trial participants self-reporting abstinence, respectively. Among those returning the readings, 12 (80.0%) reported they used the device correctly, 14 (93.3%) that it was easy to use, and 15 (100.0%) that it was acceptable.

There were no statistically significant differences between participants returning the CO tests or not with respect to baseline characteristics or the study arm, except for those who returned the CO readings being more likely to had used electronic cigarettes before (40.9% vs. 73.3%, *p* = 0.04; see Table 1). Participants with known app device system (Android: 35%, iOS: 22.7%) had marginally greater proportion of results returned than those with device status Unknown (17.0%). A significantly greater proportion of participants reporting abstinence via the app (53.8%) sent their readings in comparison to those reporting it via e-mail (0.0%), or phone (21.1%) (*p* = 0.01). There were no statistically significant differences between those who returned CO readings, and those who did not return them, on other trial outcomes (see Table A1).

**Table 1:** Baseline characteristics of BupaQuit trial participants who self-reported not smoking and who returned or did not return their CO readings.

	Total (n = 59)	Did not return CO readings (n = 44)	Returned CO readings (n = 15)	*p*^a^
Study arm in BupaQuit trial, %(N)				
Intervention	42.4 (25)	40.9 (18)	46.7 (7)	0.77
Control	57.6 (34)	59.1 (26)	53.3 (8)	
Age (years) Mean (SD)	33.0 (10.6)	33.1 (10.9)	32.7 (10.0)	0.90
Smokes within 5 min of waking up % (N)	20.3 (12)	18.2 (8)	26.7 (4)	0.48
Confidence to stop (1–7) Mean (SD)	4.9 (1.4)	4.8 (1.4)	5.3 (1.3)	0.17
Female % (N)	32.2 (19)	29.5 (13)	40.0 (6)	0.53
Occupation % (N)				
Manual	55.9 (33)	54.5 (24)	60.0 (9)	0.92
Non-manual	20.3 (12)	20.5 (9)	20.0 (3)	
Other, incl. retired, unemployed, student	23.7 (14)	25.0 (11)	20.0 (3)	
Has post-16 years qualification % (N)	74.6 (44)	77.3 (34)	66.7 (10)	0.50
Strength of urges (0–5) Mean (SD)	2.8 (0.8)	2.8 (0.8)	2.7 (0.8)	0.80
Made an attempt to quit last year % (N)	59.3 (35)	54.4 (24)	73.7 (11)	0.24
Stopped smoking for >1 week % (N)	84.7 (50)	81.8 (36)	93.3 (14)	0.42
Recruitment channel				
Advertisement on Twitter/Facebook	33.9 (20)	29.5 (13)	46.7 (7)	0.42
App store searches	30.5 (18)	34.1 (15)	20.0 (3)	
Other (email, word of mouth, poster)	35.6 (21)	36.4 (16)	33.3 (5)	
Restricted phone access during the day % (N)	22.0 (13)	25.0 (11)	13.3 (2)	0.48
Used any cessation aids in the past^b^ % (N)				
No aids	22.0 (13)	25.0 (11)	13.3 (2)	0.48
Stop smoking services	32.2 (19)	31.8 (14)	33.3 (5)	1.00
Medications	50.8 (30)	52.3 (23)	46.7 (7)	0.77
E-cigarettes	49.2 (29)	40.9 (18)	73.3 (11)	0.04
Apps	13.6 (8)	13.6 (6)	13.3 (2)	1.00
Other incl. websites and quitline	23.7 (14)	20.5 (9)	33.3 (5)	0.32
Smartphone operating system				
Android	33.9 (20)	29.5 (13)	46.7 (7)	0.46
iOS	37.3 (22)	38.6 (17)	33.3 (5)	
Unknown	28.8 (17)	31.8 (14)	20.0 (3)	

a *p*-Value from Fisher's exact test for 2 × 2 tables, from chi-square test for other categorical variables, and from independent t-test for continuous variables.

b Participants could select one or more answers.

Through subsequent opportunistic communication with some of the participants (e.g. when issuing invitation to follow-up telephone interviews or at 6 month follow-up) and thanks to one parcel being returned to the office, eight of the 44 missing CO readings were accounted for: one incorrect address, one participant was unable to accept large packages, one lost the device, one had forgotten about the test, one refused to email readings and share them with Bupa seeing it as an intrusive procedure, and three had no access to a Windows PC.

## 4 Discussion

Remote assessment of self-reported abstinence in trials of digital cessation interventions using personal CO monitors is a promising and, in theory, more attractive and cheaper alternative to other available methods. However, in this study only a quarter of participants provided results of biochemical verification, much fewer than around 60–80% observed in other studies (e.g. (Glasgow et al., 1993), and unpublished data from (Brown et al., 2014)). These findings suggest that using CO monitors that connect to computers to remotely assess abstinence in a smartphone-based cessation trial was not feasible as per this study's protocol. Lack of reimbursement or no contact with the researcher at enrolment could be among possible reasons, suggesting that for the purpose of follow-up in trials, CO testing may not be a feasible substitute for traditional methods, such as saliva testing, without implementing additional procedures (e.g. incentives).

Indeed, studies that observed better engagement with remote CO testing adopted very different procedures in comparison with the present study, and often implemented CO testing during study initiation and as an integral part of quitting or cutting down, rather than only as a method to validate self-reported abstinence (Dallery & Glenn, 2005; Hertzberg et al., 2013; Karelitz et al., 2017). For example, such studies involved training with a researcher on using the CO device, offered incentives (e.g. cash or vouchers) for reporting any CO readings or for meeting certain CO thresholds, required or encouraged regular and video-recorded CO testing (e.g. daily, or twice daily), offered additional cessation resources (e.g. a website, pharmacotherapy, behavioural support), and involved other regular contact with the researchers (e.g. remote monitoring of the readings to identify falsifications, or lab-visits) (Dallery & Glenn, 2005; Hertzberg et al., 2013; Karelitz et al., 2017). Finally, some studies also used a more expensive CO device (e.g. piCO Smokerlyzer developed by Bedfont for clinical use) (Dallery & Glenn, 2005; Karelitz et al., 2017), which could have nontrivial impact on the experience of testing itself.

Among the main challenges in using CO monitors in this trial were lack of affordable and practical means to trace package delivery, retrieve unused devices, and to systematically collect feedback or reasons for the missing results. Some of the underlying causes were common with other trials of digital interventions, including limited contact with participants, lack of a closer rapport or accountability, and little opportunity to discuss procedures (Brown et al., 2014). Several practical barriers to CO testing were also identified, although most cases remained unexplained. Some other possible reasons could include: (a) lack of reimbursement (retaining CO monitors might not be a sufficient incentive), (b) participants unavailable at the address provided – we were able to confirm only two-thirds of addresses, (c) having used the CO test but not sharing the results with the researchers, (d) over-reporting of abstinence at follow-up, and not willing to share CO result confirming smoking, and (e) low commitment to the study or limited app engagement. The latter is further supported by the observations that the CO results tended to be returned more often by those reporting abstinence via the app, and those engaging with the app in the first place.

The study had limitations. First, it was an exploratory and observational study using secondary data from a randomized trial. Due to concerns about attrition and participant burden, a more detailed assessment of the CO testing procedure was not feasible. However, contacting participants over email or phone to collect other trial outcome data was challenging, and it is unlikely that attempts at collecting further data on CO test would be fruitful. Second, the PC-based COmpactUSB™ Smokerlyzer® model used in this study has been discontinued and replaced by a model that connects to Android/iOS smartphones and tablets (iCO™ Smokerlyzer®). Smartphone-enabled CO monitoring devices (Meredith et al., 2014) might be more accessible and thus could increase the proportion of CO tests returned in future studies of cessation apps. The feasibility of using these devices requires further research, but the observations from this study should nevertheless apply to other settings when the CO devices are posted as part of the follow-up and verification of abstinence.

In the present study, the CO monitors were posted to participants only once the follow-up had started, and to be used by them only at one time point. Among the key benefits of using personal CO monitors is that they allow for repeat testing and monitoring of progress in quitting (i.e. demonstrating a decline in ppm values from baseline), which this study has not explored. Providing participants with CO monitors at the start of the trial or at the initiation of a quit attempt could increase acceptability and engagement with the devices, as well as cessation outcomes (Beard & West, 2012; Dallery & Glenn, 2005; Shahab et al., 2011), which should be assessed in future research. Additionally, future studies could involve experimental designs, e.g. a head-to-head comparison of several methods of abstinence validations, to better asses the acceptability and feasibility of the individual tests.

The findings suggest that studies using remote CO testing to validate abstinence in trials may require separate reimbursement and establishing a better rapport with participants, as well as using software that enables recording of installation or initiation of device use without intrusive collection of non-trial data.

## Appendix A Supplementary data

Appendix A1: The letter about CO testing and instructions sent to participants. Fig. S1: Screenshots of software accompanying Bedfont® COmpactUSB™ Smokerlyzer® in BupaQuit Trial. Table A1: Follow-up channels, satisfaction and app usage among a sub-sample of BupaQuit trial participants, who self-reported not smoking and who were posted CO monitors (n = 59). Image 1
