# Supplementary material for: Lessons learned from unsuccessful use of personal carbon monoxide monitors to remotely assess abstinence in a pragmatic trial of a smartphone stop smoking app – A secondary analysis
Source: Addict Behav Rep. 2018 Jul 23;9:100122. doi: 10.1016/j.abrep.2018.07.003 (PMC6542188; doi:10.1016/j.abrep.2018.07.003)
Supplement: Supplementary file 1 — Appendix A1: The letter about CO testing and instructions sent to participants. Fig. S1: Screenshots of software accompanying Bedfont® COmpactUSB™ Smokerlyzer® in BupaQuit Trial. Table A1: Follow-up channels, satisfaction and app usage among a sub-sample of BupaQuit trial participants, who self-reported not smoking and who were posted CO monitors (n = 59). [file mmc1.docx]

# Lessons learned from unsuccessful use of personal carbon monoxide monitors to remotely assess abstinence in a pragmatic trial of a smartphone stop smoking app – A secondary analysis.

# Appendix - Supplementary materials

Aleksandra Herbec^1,2^, MSc, Jamie Brown^1,2^, PhD, Lion Shahab^1,2^, PhD, Robert West^1,2^, PhD

^1^ Department of Behavioural Science and Health, University College London, London, UK

^2^ UCL Tobacco and Alcohol Research Group (UTARG), UK

**Trial registration:** ISRCTN10548241 (<http://www.isrctn.com/ISRCTN10548241>)

#

**Appendix A1:** Letter about CO testing and instructions sent to Participants Part 1^[[1]](#footnote-1)^


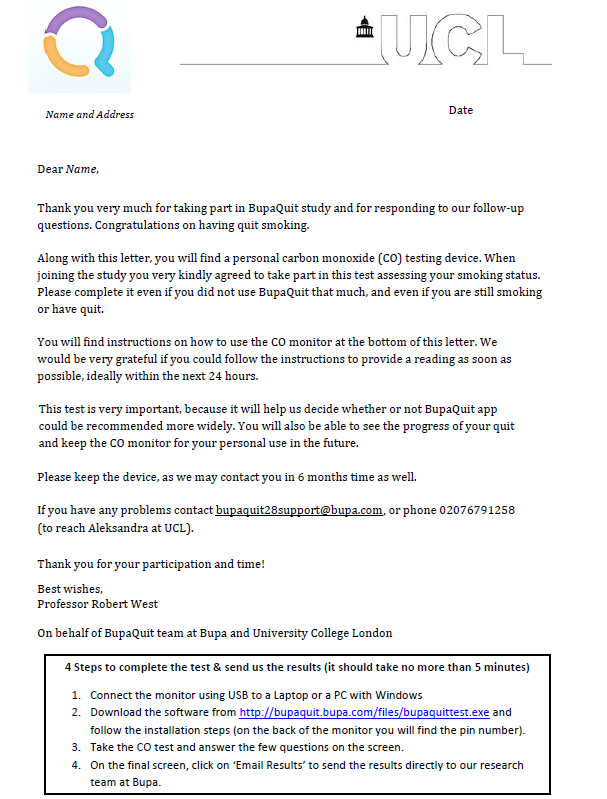


**Appendix A1 (cont.):** Letter about CO testing and instructions sent to Participants Part 2


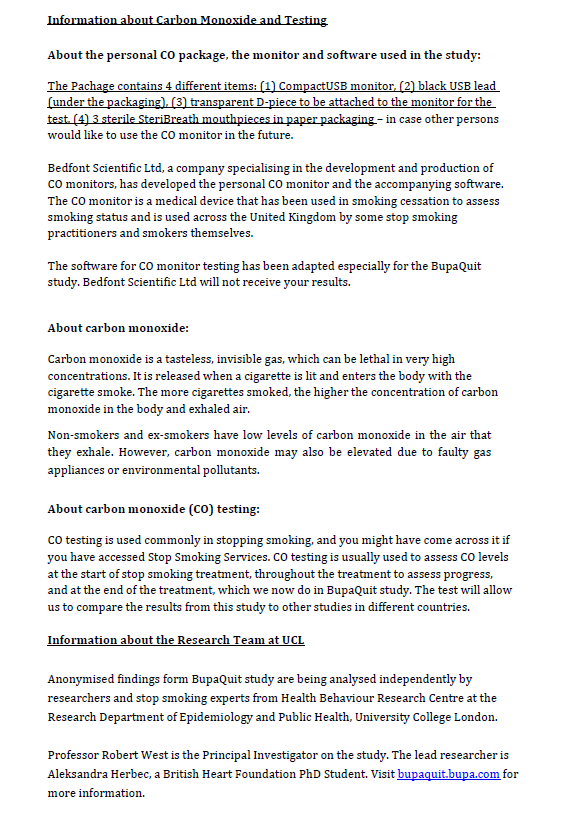


**Appendix A1 (cont.):** Letter about CO testing and instructions sent to Participants Part 3


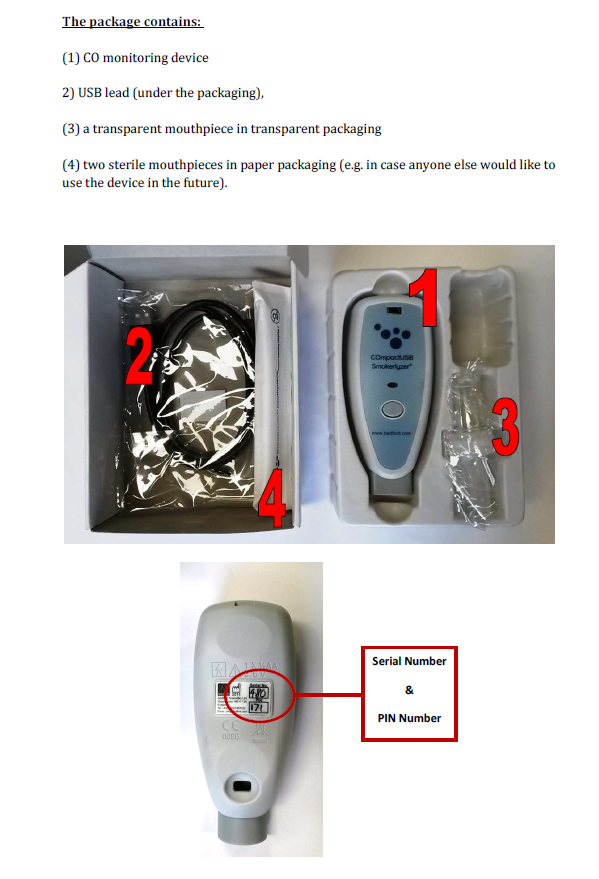


# Figure A1: Screenshots of software accompanying Bedfont® COmpactUSB^TM^ Smokerlyzer® in BupaQuit Trial

Screenshots of software accompanying personal CO monitors – the original software accompanying COmpactUSB^TM^ Smokerlyzers® was adapted by Bedfont® for the BupaQuit trial. The changes involved:

Step 1: The two original questions comprising Heaviness of Smoking Index were replaced with four additional questions that were supporting data collection for the trial and were used to evaluate the use of CO monitor.

Step 2: changing the tailored feedback.


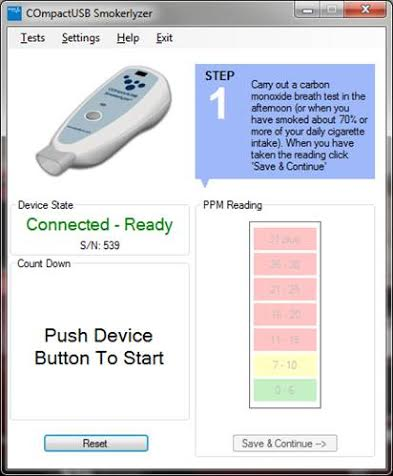

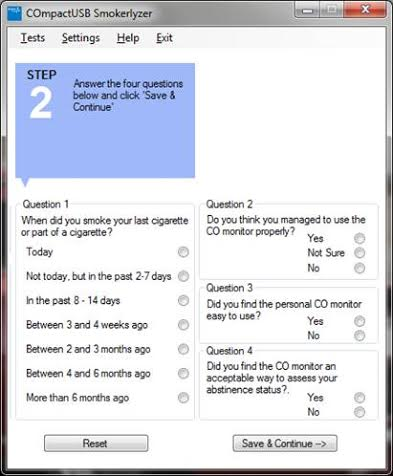

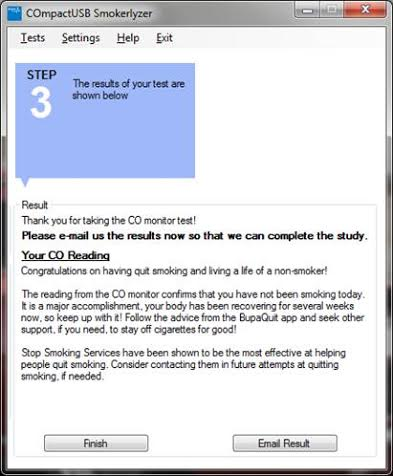


*Reproduced with permission from Bedfont® Scientific Ltd*

**Table A1:** Follow-up channels, satisfaction and app usage among a sub-sample of BupaQuit trial participants, who self-reported not smoking and who were posted CO monitors (n=59).

|  | **Did not return CO readings**  **(n=44)** | **Returned CO readings**  **(n=15)** | ***p*** |
| --- | --- | --- | --- |
| **Follow-up channel for primary outcome at 4 weeks % (n/N)** | |  |  |
| App | 13.6 (6) | 46.7 (7) | **0.01** |
| Email | 18.2 (8) | 0.0 (0) |  |
| Phone | 68.2 (30) | 53.3 (8) |  |
| **Satisfaction (available for a sub-sample who answered additional questions)** | **n=9** | **n=8** |  |
| Would use the app in the future | 77.8 (7) | 100.0 (8) | 0.47 |
| Would recommend the app to others | 77.8 (7) | 100.0 (8) | 0.47 |
| **App usage classification^§^** |  |  |  |
| Accessed only pre-quit app | 4.5 (2) | 0.0 (0) | 0.57 |
| Accessed only post-quit app | 27.3 (12) | 26.6 (4) |  |
| Accessed both pre- and post-quit app | 36.4 (16) | 53.3 (8) |  |
| Not engaged with the app | 31.8 (14) | 20.0 (3) |  |
| **Engaged with the app** | 68.2 (30) | 80.0 (12) | 0.52 |
| **Usage data available (during trial only) % (n/N)** | **n=30** | **n=12** |  |
| Total logins, Median (IQR)  Mean (SD)^a^ | 8.5 (21.0) | 15.5 (55.0) | 0.22 |
|  | 21.5 (29.40) | 35.8 (41.73) | 0.21 |
| Total time (sec)^¥^ Median(IQR) | 256.5 (528.0) | 538.0 (1002.25) | 0.77 |
| Mean (SD)^a^ | 601.0 (800.0) | 66.925 (655.04) | 0.79 |
| Time per login (sec) ^¥^ Median(IQR) | 34.3 (33.0) | 20.6 (44.23) | 0.73 |
| Mean (SD)^a^ | 36.2 (20.05) | 31.2 (28.20) | 0.61 |
| **App usage classification^§^** |  |  |  |
| Accessed only pre-quit app | 4.5 (2) | 0.0 (0) | 0.57 |
| Accessed only post-quit app | 27.3 (12) | 26.6 (4) |  |
| Accessed both pre- and post-quit app | 36.4 (16) | 53.3 (8) |  |
| Not engaged with the app | 31.8 (14) | 20.0 (3) |  |

^¥^Total time, excluding registration, from the first use until follow-up. The time spent is an underestimate: (a) data from offline app use save locally on user’s device but would not synchronize if users had not accessed the app while being online on a future occasion; and (b) the interaction between an app page and the server occurs when a page is loaded. No further communication with the server occurs until another page is loaded. Hence, it is not possible to identify the exact duration of the last interaction when it ends with exiting the app. ^§^Only assessed among the sample with usage data available. Pre-quit app use only means that participants set the quit date in the future and accessed only pre-quit content; only the post-quit intervention app offered craving aids. ^a^ we provide Means to enable comparison with other studies. However, the usage data were skewed and hence we conducted and report results from non-parametric

1. After several CO letters were sent, we introduced some small changes to subsequent letters, e.g. wording to emphasise that CO results should be returned even if participants have not used the app much or if they have been smoking. [↑](#footnote-ref-1)
